# Social prescribing as a tool for integrated care—insights from a cross-sectional study with German general practitioners

**DOI:** 10.3389/fpubh.2026.1757075

**Published:** 2026-05-13

**Authors:** Dora E. Haxhija, Michael A. Paultisch, Christina Radl-Karimi, Jennifer Engler, Andrea Siebenhofer

**Affiliations:** 1 Institute of General Practice, Goethe University Frankfurt, Frankfurt am Main, Germany; 2 Institute of General Practice and Evidence-based Health Services Research, Medical University of Graz, Graz, Austria; 3 Gesundheitsamt Frankfurt am Main (Public Health Authority), Frankfurt am Main, Germany

**Keywords:** general practitioners, health promotion, implementation, integrated care, primary care, social prescribing

## Abstract

**Introduction:**

Social prescribing (SP), a patient-centered approach integrating non-medical interventions into primary care to address social determinants of health, is relatively unknown in Germany. This study explores German general practitioners’ (GPs) awareness and perspectives on SP to assess its potential integration into healthcare.

**Methods:**

A cross-sectional survey among German GPs evaluated their familiarity with SP, perceived effectiveness, willingness to adopt it, and potential barriers to implementation. Surveys were analyzed using descriptive and interferential statistics. With a response rate of 7.9% 101 questionnaires were completed.

**Results:**

Most GPs were unfamiliar with the term SP, yet the majority expressed willingness to adopt it and some had already engaged in SP-related activities, even if unknowingly. GPs often encountered social issues such as mental strain, workplace-related stress, caregiving for relatives, and loneliness, which they believed could be effectively addressed through SP. SP usage significantly influenced perceived benefits and barriers, including the lack of time during consultations, lack of knowledge about referral structures and funding additional workforce. Almost all viewed it as beneficial for improving patient care, with most preferring outsourcing SP. GPs practicing in towns with a population under 5,000 were significantly more likely to adopt SP compared to the broader sample.

**Discussion/conclusion:**

Participating GPs are largely supportive of SP and believe it has the potential to improve patient outcomes and reduce strain on the healthcare system, but lack practical implementation. We believe that future initiatives should focus on educating healthcare providers and integrating SP into primary care policies to maximize its impact on healthcare sustainability and equity.

## 1 Introduction

### 1.1 Background

General practitioners (GPs) are often the first professional point of contact for patients with both medical and social concerns (1), ranging from loneliness to financial difficulties and workplace challenges (2). Historically, social stressors and socioeconomic factors have not received sufficient attention in regular healthcare, despite their significant impact on individual health (3). Social prescribing (SP) offers a structured, holistic, patient-centered approach to adequately support patients by connecting them to non-medical services within their local communities (4).

It is estimated, that in the UK, where SP originated, 20% of GP consultations are linked to social issues, while in Germany this varies from 14 to 25% (2, 5). With the demographic shift and the rising incidence of chronic diseases, GPs are increasingly reaching their capacity limits (6), particularly in managing patients with low socioeconomic status, who have shown to be in poorer overall health and face higher prevalences of chronic conditions such as diabetes, heart disease, lung disease, and stroke (7). Moreover, the aging population is becoming more vulnerable to social determinants of health. Addressing the needs of these complex patients requires more time (8) and resources that may extend beyond the expertise available within general practice (9, 10).

SP includes a range of models. At one end are GP-led models, in which physicians address patients’ social needs within routine consultation. These include model 1: signposting and model 2: direct referral (11). Such approaches largely rely on limited consultation time and patients’ own initiative. SP ideally involves trained personnel, known as link workers (LWs), who assess patients’ social, emotional, and/or practical needs and connect them to suitable community-based programs after being referred by a GP (12). In model 3: link-worker-based SP, LWs receive referrals from GPs, conduct individualized assessments and coordinate access to relevant services (11). This approach emphasizes preventive health measures, with collaborative development and active patient participation in designing their personalized care as key elements (13, 14). Beyond these pathways, SP may also adopt holistic models, including model 4: holistic and individual-based SP and model 5: holistic and group-based SP, in which referrals can come from other care organizations—not strictly from the GPs (11).

SP has the potential to improve overall health and well-being, while reducing the workload on healthcare providers and decreasing demand for medical services (15). It has shown to reduce the number of unnecessary GP visits and hospital appointments in the UK by 28%, thereby alleviating the burden on individual healthcare professionals (16, 17). Since its initiation in the UK in the 1990s, SP has become a key component of the Universal Personalised Care (UPC) project, rolled out for 2023–24, highlighting its importance in modern healthcare (18). While SP has gained international recognition, including in Austria, where pilot programs were introduced in 2021, in neighboring Germany it remains in its early stages with limited awareness and engagement among healthcare providers (13, 19).

While numerous studies have explored doctor-patient interactions within existing SP programs (20), the viewpoints of GPs in Germany, where such programs are not yet systematically implemented, are still not well understood. Stumm et al. (21) showed that there was a significant lack of awareness of already established community care points among German GPs. Similarly, another study demonstrated the lack of inter-professional collaboration between social care and general practice, which hinders proper management of patients’ social problems for GPs in Germany (22). Only recently, a large-scale study has addressed different approaches of dealing with patients’ social issues in Germany, surveying over 1,000 GPs. They compared meaningfulness and usage-wish of four different non-medical services in primary care, being SP, in-practice social work services, health kiosks and integrated primary care centers. While more the 65% of the GPs wanted to use one of these models, there was no specific model that the majority preferred (23). Beyond the German context, trust in GPs and respect for their professional authority have been identified as crucial factors to positively influence patient adherence to SP interventions (12), which emphasizes the need to understand GP views on SP and its’ implementation early on.

### 1.2 Objective

This study aims to address gaps in knowledge regarding the potential implementation of SP across Germany. Specifically, it seeks to determine whether the SP concept is known to GPs, to understand their attitudes toward it and to assess their willingness to integrate it in some way. Additionally, our aim was to explore its applicability to common issues faced in clinical practice. The study examines benefits and potential barriers to its implementation, analyzing how GP characteristics relate to their SP-like usage. By answering the following research questions, this study seeks to create a view of GPs minds in order to promote overall future efforts to effectively integrate SP into daily clinical practice in Germany:

1. Is the term SP familiar to German GPs and to what extent is SP currently being used?
2. Is SP considered an effective tool for addressing the social problems of patients encountered by GPs?
3. Would GPs like to practice SP and under which circumstances could SP be implemented in Germany?
4. Do GPs think that patient care can be improved through SP?
5. Are sociodemographic and practice characteristics as well as specific barriers and benefits associated with SP usage?

## 2 Materials and methods

We conducted a cross-sectional survey among German GPs between July and December 2023. A 29-item questionnaire was developed following an extensive review of literature: 17 questions addressing SP and 12 questions addressing the sociodemographic characteristics of the participants. A preliminary pilot test was conducted with several GPs, using the think-aloud method, resulting in minor refinement of specific questions. Additionally, three independent GPs reviewed the questionnaire for comprehensibility (see Supplementary material 1).

GPs who were invited to participate, were either contacted via coordinators of their primary care research network or via email. GPs from 10 federal states in Germany were contacted: Bremen, Hamburg, Hesse, Mecklenburg-Western Pomerania, Lower Saxony, Rhineland-Palatinate, Saarland, Saxony, Saxony-Anhalt, Schleswig-Holstein. In case of direct contact with the GPs, email addresses were sourced from publicly accessible lists provided by statutory health insurance associations, and invitations included a cover letter explaining the study’s objective along with the link to the online questionnaire. A reminder email was sent after 3 months to encourage participation. The total number of emails sent amounted to 1,595. Further, a flyer including the study’s objective and a QR code linking the survey, was presented at the 57th congress of the German Society of General Practice and Family Medicine in Berlin.

### 2.1 Ethical considerations

As the study did not involve any intervention in clinical practice of the GPs or collection of patient or identifiable personal data, an all data were collected anonymously, an ethics vote was deemed unnecessary. The study was conducted in accordance with data protection regulations [EU GDPR 2016/679, Recital 26 and Article 4 (1)] (24). Participation was voluntary.

### 2.2 Analysis

To answer the research questions, several quantitative analytical approaches were applied:

For research questions 1–4, descriptive statistics and visualization methods were employed. A correlation analysis was performed in order to explore the perceived effectiveness of SP in addressing the specific social issues encountered by GPs every day. It provides insight into whether the social issues GPs believe are most responsive to intervention through SP are also those that are most frequently seen in practice.

For research question 5, a multi-step approach was chosen:

1. Potential differences of SP usage in respect of given barriers and facilitators as well as associations of SP usage with given sociodemographic and practice-specific characteristics were checked by exploratory data analysis (EDA) (inspection of Box Whisker-Plots).
2. Magnitude and relevance of those potential differences and associations were assessed by effect sizes.
3. Relevant differences and associations were tested for statistical significance (*α* = 5%).

When in EDA potential and apparent differences of barriers and facilitators were detected, they were further inspected by Cliff’s Delta (*δ*) to assess if those differences had a relevant size: Differences and barriers with an at least small size (> 0.15) were retained for further analyses of practically meaningful differences (25, 26). These facilitators and differences were tested on statistical significance (to test transferability of the sample’s results to population) by non-parametric multivariate analysis of variance (PERMANOVA), if their correlations with each other did not exceed *ρ* = 0.5 to avoid multicollinearity (27). The PERMANOVA was performed to evaluate the collective relationship between SP usage (independent variable) and ordinal-scaled barriers and facilitators (dependent variables). Cliff’s delta und PERMANOVA were chosen because of the ordinal nature of the measurements of facilitators and barriers. For PERMANOVA the Manhattan metric was used, as it is suitable for ordinal data (28, 29).

This two-step approach—filtering variables by effect size before multivariate testing for statistical significance—reduces the risk of overfitting and ensures that only variables with substantive associations are included in the final model (30).

Sociodemographic and practice-specific differences (independent variables: sex, age, practice location) were further examined by means of binary logistic regression (stepwise backward), employing odds ratios (ORs) as effect sizes. Ordinal variables (practice location), were dummy-coded enabling regression analysis. The group mirroring sample-wide proportions of SP-usage, was selected as the reference category to anchor comparisons, ensuring that regressions coefficients for the other groups reflect deviations relative to the most representative group. This approach aligns with recommendations for selecting reference categories that reflect population-level characteristics to improve interpretability (31, 32).

Quantitative analyses were performed in R-Studio (Version 2023.12.1 + 402) using tidyverse (dplyr, tidyr and ggplot for preprocessing and visualization) (33), GUniFrac (function adonis3 for non-parametric MANOVA) (34), stats (function glm for binary logistic regression and odds ratios) (35), and effsize (for Cliff’s *δ*) (36).

## 3 Results

The survey was conducted from 7 July 2023 to 4 December 2023 and received a response rate of 7.9% (126 responses), with 101 complete questionnaires available for analysis after excluding incomplete responses. The majority of GPs were male (54.5%) and ran a single-handed practice (49.5%). Academic vs. non-academic practices were almost equally represented. All characteristics of the participating GPs are detailed in Table 1.

**Table 1:** Sociodemographic characteristics of participating GPs.

Demographics	*n =* 101	%
Sex		
Female	46	45.5
Male	55	54.5
Age (years)		
< 35	4	4.0
35–45	14	13.9
46–55	24	23.8
> 55	59	58.4
Practice type		
Single handed	50	49.5
Group practice	30	29.7
Community health center (MVZ)	21	20.8
Size of town of practice		
< 5,000	20	19.8
> 5,000–20,000	30	29.7
> 20,000–100,000	25	24.8
> 100,000	26	25.7
Academic practice		
Yes	60	59.4
No	41	40.6
Years in practice		
< 5	14	13.9
5–15	22	21.8
16–30	40	39.6
> 30	25	24.8
Patients per quartal		
< 750	10	9.9
750–1,500	35	34.7
1,500–2,500	40	39.6
> 2,500	16	15.8
Federal state		
Baden-Wuerttemberg	2	2.0
Bavaria	2	2.0
Bremen	3	3.0
Hamburg	7	6.9
Hesse	59	58.4
Mecklenburg-Western Pomerania	7	6.9
Lower Saxony	3	3.0
North Rhine-Westphalia	2	2.0
Rhineland-Palatinate	9	8.9
Saarland	4	4.0
Schleswig-Holstein	3	3.0

Sociodemographic characteristics of the 101 participants of our survey.

### 3.1 Objective 1: familiarity and usage of SP

Prior to participation, 84% of GPs reported that they were not familiar with the term SP. After being introduced to the concept within the questionnaire by a practical example of SP, 35% of GPs said that they had implemented SP-related activities in their practice.

Out of the 35% that had already practiced some form of SP, 70–85% of GPs said that they had offered GP-led SP approaches, such as: *own consultation in the practice during a personal conversation with the patient, referral to institutions to which the patient initiates contact* and *providing flyers/addresses to patients*. Almost 50% had referred patients directly to external support services, while structured LW-based models were rarely used. Fewer than 5% had referred their patients to external link workers and even fewer had an internal link worker in their practice.

### 3.2 Objective 2: effectiveness of SP

GPs were first asked to answer regarding the frequency (*very often, often, rarely, never*) of 12 social problems in practice, such as loneliness, housing difficulties, or mental health concerns. Secondly, they had to rate the perceived impact (*high, moderate, low, none)* SP could have on the previously mentioned social problems.

Social issues seen most in practice were *mental strain/overwhelm* (65% very often, 31% often) and *workplace burdens/unemployment* (60% very often, 36% often), followed by *caregiving for relatives* (32% very often, 51% often), *loneliness* (19% very often, 63% often), *illness or death of relatives or friends* (20% very often, 55% often) and *dispute with a close person* (18% very often, 60% often). Issues such as *discrimination/exclusion*, *abuse/domestic violence*, *problems with housing/homelessness* and *issues in education/training* were infrequently encountered (see Supplementary Figure 1).

The perceived benefit of SP was particularly high for issues related to loneliness, mental strain and caregiving for relatives. Specifically, 82% of GPs rated SP as highly effective for reducing *loneliness*, while 54% viewed it highly effective for dealing with *caregiving for relatives* and 54% viewed it highly effective for addressing *illness or death of relatives or friends* (see Supplementary Figure 2).

**Figure 1:**
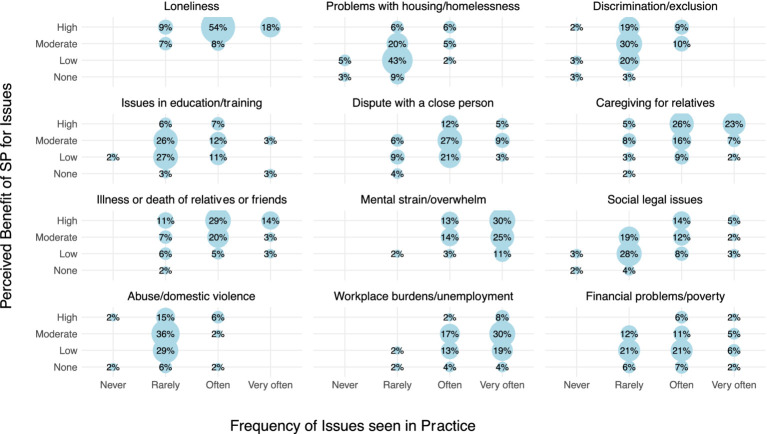
Correlation between issue frequency and SP impact perception among GPs. Correlation analysis highlighting the frequency of social problems encountered in practice (shown on x-axis) and the corresponding perceived effectiveness of SP (shown on y-axis) in coping with these issues. Larger dots represent a higher percentage of GPs expressing the same combinations of views. The clustering of dots in the upper right quadrant of each of the 12 subfigures indicates social issues that are both frequently encountered and perceived as highly susceptible to intervention via SP.

While loneliness and mental health were frequently seen as issues where SP could have a substantial impact, fewer GPs believed SP would be as effective for addressing financial problems or workplace burdens, despite these issues also being commonly encountered. The visual representation is shown in Figure 1.

### 3.3 Objective 3: implementation of SP

Currently, 68% of GPs are unsatisfied with their ability to help patients with their social concerns when they present in practice and 93% believe that SP could be a useful tool to enhance patient care. Almost all GPs (94%) expressed the willingness to adopt SP within their practice.

The preferred referral method would be *referring patient to institutionalized contact person or link worker* (56% fully, 32% somewhat), followed by *providing information or addresses of different institutions the patient would then contact on their own* (46% fully, 46% somewhat), *referral to telephone consultation services the patients would then contact independently* (45% fully, 40% somewhat) or *referring patients to an external link worker* (46% fully, 35% somewhat).

Mixed responses, slightly more positive, were observed for online platforms listing SP activities and referral hotlines with the physician establishing contact. Less favored were medical assistant or nurses functioning as link workers and the establishment of direct contact between physicians and institutions.

### 3.4 Objective 4: potential of improvement through SP

GPs perceived SP as beneficial for patient care and wellbeing. 43% strongly and 42% moderately agreed that SP could *enhance overall patient care*, while 44% strongly and a further 49% moderately agreed that it could *increase patient satisfaction*. Furthermore, 62% strongly agreed that SP could *alleviate loneliness*, with 31% moderately agreeing and 3% perceiving no potential effect. The perceived *impact on the mental health of patients* was positive with 56% of GPs strongly and 36% moderately agreeing.

Regarding healthcare utilization, 42% of GPs strongly and 27% moderately agreed that SP could *reduce consultations with other healthcare professionals*, while 46% strongly agreed that it could *decrease consultations within their own practice*. 45% felt SP could *lower medication prescriptions*, though 19% saw no impact. Perceptions on *reducing practice workload* were more divided with 34% strongly and 34% moderately agreeing, while 24% reported no expected effect (see Supplementary Figure 3).

### 3.5 Objective 5: differences in SP usage based on GP characteristics, barriers and benefits

Exploratory data analysis revealed SP-usage differences appeared especially pronounced among GPs working in smaller towns and among male GPs. In addition, barriers such as *lack of time during consultations, lack of knowledge about available regional service*s were associated with less self-reported SP-usage, whereas the perceived facilitator *reducing the number of medication prescriptions* was associated with a higher rate.

Out of eight benefits and nine barriers (Supplementary Figures 3, 4), after inspection of EDA-results, four benefits and five barriers were further analyzed in terms of effect sizes. Medium effect sizes were observed for barriers such as *lack of time during consultations* (*δ* = 0.34) and *lack of knowledge about available local services* (*δ* = −0.35), as well as for the benefit of *reducing the number of medication prescriptions* (δ = 0.37). Smaller effect sizes were noted for barriers including *funding for additional staff* (δ = 0.20) and *lack of structures for referrals* (δ = −0.17). Spearman’s rank correlation revealed moderate associations between the five included variables, with the strongest correlation observed between lack of structures for referrals and lack of knowledge about available local services (*ρ* = 0.47). None exceeded ρ = 0.5, suggesting low multicollinearity and supporting their inclusion in multivariate models. Identified relevant differences of barriers and facilitator in respect of SP usage were statistically significant (*p* = .034, R^2^ = 0.03), visible in Supplementary Table 2. Differences of usage were observed as follows: *lack of time during consultation* (less SP usage), *funding for additional staff* (higher SP usage), *lack of knowledge about available regional services* (less SP usage), *lack of structures for referral* (less SP usage) and *benefit of reducing numbers of medication prescriptions* (higher SP usage).

Binary logistic regression was conducted, retaining gender and practice location as relevant predictors, in order to assess whether the adoption of SP could be explained by sociodemographic and practice-specific characteristics. GPs practicing in towns with fewer than 5,000 inhabitants were significantly more likely to adopt SP compared to the reference group (OR = 3.024, CI 1.13–8.13, *p* = .028), while male GPs showed a trend toward higher adoption, though not reaching statistical significance (OR = 2.114, CI 0.88–5.06, *p* = .09) (see Supplementary Tables 2, 3).

## 4 Discussion

This study demonstrates that while most participants are unfamiliar with the term SP, some are already performing SP-like activities, and there is a strong willingness to implement SP formally. This lack of awareness aligns with previous findings in Germany, that reported a similar lack of familiarity with community care points among German GPs (21). The discrepancy between terminology and practice mirrors experiences in other European countries during early SP adoption phases; such as, for instance, in Austria’s pilot programs, GPs initially lacked familiarity with the term “social prescribing” but recognized its alignment with holistic care principles once contextualized (19).

Most participants expressed dissatisfaction with their ability to address patients’ social concerns, emphasizing the need for integrated care solutions, as shown by Zimmermann et al. (22), who noted that GPs often feel ill-equipped to manage the social determinants of health effectively. Our study further revealed that the participating GPs spend a significant portion of their time addressing social issues, with 55% reporting that they encounter social problems more than three times daily and 51% dedicating more than 20% of their working hours to these concerns. These findings align with prior research, which found that social issues play a role in 20–25% of consultations, often consuming a substantial portion of appointment time (2, 40). Austrian evaluation findings demonstrate that through link worker integration SP reduces physician workload while increasing available time for patient care (41). This underscores the need for interventions like SP in Germany to alleviate the burden on GPs and improve patient outcomes.

Despite limited awareness of the term SP, 35% of participants reported engaging in SP-like activities. These included *providing patients with flyers or addresses*, corresponding to model 1: signposting, as well as *referrals to third-sector organizations* (model 2: direct referral). Additionally, some GPs reported *referring patients to institutions that patient would contact on their own*. While this does not align with a specific defined SP model, it can be classified as a form of signposting. However, fewer than 3% used formal link workers, as this system is not yet implemented in Germany. This corresponds with Zimmermann et al. (22), who found that GPs often rely on informal methods, such as direct consultations or providing information, rather than structured SP programs. When asked about their preferred methods for implementing SP, most respondents favored *outsourcing to institutionalized or external link workers* or *providing patients with contact information for external services*. These findings suggest that while GPs are open to SP, they prefer outsourcing SP, which aligns with successfully implemented programs in the UK, where it has been shown that GPs preferred delegating social needs to LWs and that structured SP programs with dedicated LWs led to better patient outcomes (14). This is promising for the potential success of SP implementation, provided that the wish to outsource SP can be generalized to all German GPs rather than just our small study group. Herrmann and Napierala (23), who had a higher response rate and bigger study population, recently also demonstrated that the majority of German GPs preferred outsourcing solutions over direct service provision. In a further analysis of their data, perceptions regarding which care model should be implemented on a broader scale in Germany were assessed. In that study most participants selected SP (35%), followed by in-practice social work services (32%) (37) This shows promising results concerning a more generalized SP adoption. While their study compared four different integrated care models, our research specifically examined the nature of social problems encountered in primary care, the perceived effectiveness of SP in addressing them, and the barriers and benefits associated with its implementation.

Although only a portion of respondents currently incorporate some way of SP in their routine care, it is promising that the concept of SP—connecting patients to non-medical forms of support—is already being applied, even if not explicitly recognized as such. Formalizing these efforts into structured SP programs in Germany, as seen in the UK’s Universal Personalised Care project (18) and in Austria would be essential (19). The delegation of SP to external sources could alleviate time constraints, a barrier cited by most respondents. This resonates with the UK’s link worker model, where non-medical staff manage social referrals, allowing GPs to focus on clinical care (12). However, successful outsourcing requires robust inter-sectoral partnerships, which have historically been fragmented in Germany’s decentralized healthcare system (22).

Participating GPs identified loneliness, mental health concerns, and caregiving for relatives as the most common social issues in their practice, and they perceived SP to be potentially highly or moderately effective in addressing these problems. These findings are consistent with Polley et al. (5) and Napierala et al. (38), who demonstrated that SP indeed significantly improves outcomes for patients experiencing such issues (13, 38). Moreover, SP’s perceived efficacy in loneliness reduction mirrors international evidence; a meta-analysis by Pescheny et al. (12) found SP reduced loneliness scores by 32% in older adults. Participating GPs were less optimistic about SP’s effectiveness for relieving financial problems or workplace burdens, despite these issues being frequently encountered, perhaps suggesting that they view these as structural issues requiring systemic solutions beyond community interventions.

Our binary logistic regression revealed that participants in smaller towns (populations under 5,000) were significantly more likely to adopt SP than the reference group. Geographic disparities in SP adoption in our study group—with rural GPs being three times more likely to engage in SP-like activities—may reveal contextual implementation challenges. Rural practices often serve close-knit communities where GPs rely on intimate knowledge of local resources, facilitating informal referrals (42). Male GPs also showed a tendency toward SP adoption, though this did not reach statistical significance and contradicts previous findings (23).

Respondents perceived SP as highly beneficial for patient care and wellbeing, particularly in alleviating loneliness and enhancing overall patient satisfaction. These findings align with Chatterjee et al. (4), who highlighted the positive impact of SP on patient outcomes. However, perceptions of SP’s impact on reducing practice workload were more divided. Key barriers to SP implementation included lack of time during consultations, lack of knowledge about local services and insufficient funding for additional staff. These findings are consistent with Zimmermann et al. (22) and Gosch (39), who identified similar challenges in their studies. The PERMANOVA analysis showed a statistically significant relationship between self-reported SP usage and certain perceived barriers/benefits, highlighting their relevance in SP-implementation. Although the integration of SP is widely seen as time-consuming at first, it is often considered to be a time-saver in the long term, provided that appropriate structures are established, highlighting the importance of addressing these factors to facilitate SP adoption.

## 5 Strengths/limitations

This study provides valuable insights into the potential implementation of social prescribing in Germany. The stepwise combining of exploratory data analysis with the addition of effect size measures (Cliff’s Delta) and rank correlation, followed by a non-parametric multivariate analysis (PERMANOVA) ensured that the results are interpretable and meaningful, particularly given the ordinal nature of the data. These methodological choices strengthen the validity of the findings and provide a solid foundation for future research. Nevertheless, several limitations should be acknowledged. First, the reliance on self-reported data may introduce response bias. For example, GPs might overestimate their willingness to adopt SP due to social desirability bias and therefore positive results should be interpreted with caution; 84% of GPs indicating willingness to adopt SP does not translate into actual implementation. Second, the sample size, while adequate for our exploratory analysis, affects the generalizability of the results. The response rate of 7.9% is relatively low, which indicates selection bias. It is possible that GPs who are more interested in SP or social issues were more likely to participate in the survey, which may not necessarily reflect the views or practices of all GPs in Germany. Despite these limitations, the study offers important contributions to understanding SP adoption in Germany.

## 6 Conclusion

This study shows that in Germany GPs recognize the potential of SP to improve primary care through enhancing patient outcomes, increasing satisfaction, and reducing unnecessary consultations. Notably, among the respondents there is a preference for outsourcing SP to specialized link workers or external institutions, emphasizing the importance of well-defined referral pathways and robust support infrastructure. Successful implementation will, however, require addressing key barriers, such as time constraints, limited awareness of community resources, and insufficient funding.

Next, research steps could involve exploring stakeholder perspectives (e.g., policymakers, insurers) on SP implementation or assessing patient needs and preferences regarding social prescribing in Germany.

## Data Availability

The original contributions presented in the study are included in the article/Supplementary material, further inquiries can be directed to the corresponding author.
