# Supplementary material for: Social prescribing as a tool for integrated care—insights from a cross-sectional study with German general practitioners
Source: Front Public Health. 2026 May 13;14:1757075. doi: 10.3389/fpubh.2026.1757075 (PMC13212285; doi:10.3389/fpubh.2026.1757075)
Supplement: Supplementary file 1 [file Data_Sheet_1.docx]

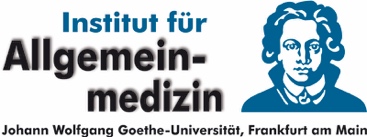
**Supplementary Material 1. Questionnaire (english translation)**

**Survey among German general practitioners regarding their view on social prescribing (SP)**

| Familiarity with SP | Yes | No |
| --- | --- | --- |
| Are you familiar with the concept of social prescribing? |  |  |

**Explanation**
Social prescribing is a treatment concept where non-medical (particularly psychosocial health-related) concerns of patients are addressed by their treating physicians. Through social prescribing, primary care providers can either refer their patients to local services themselves or through a so-called link worker. The term link worker originates from England and refers to a specialized, often external professional who provides individualized, needs- and resource-oriented support. However, the role of the link worker can also be taken on by other members of the general practice team, such as medical assistants or nursing staff.

**Example**
You, as a general practitioner, have been responsible for a patient for many years who has recently been visiting you more frequently without medical concerns. During a conversation, she tells you that her husband passed away a year ago, and since then, she has withdrawn from society. You refer the patient, using your knowledge of local services, to a suitable social support program. As a result, the patient now works at the community centre every Wednesday and you see her less often in your practice because her overall well-being has improved.

| Implementation of SP | Yes | No |
| --- | --- | --- |
| Are you already implementing social prescribing in your practice? |  |  |

| Do you carry out any of the following types of social prescribing in your practice? Multiple answers possible | Yes | No |
| --- | --- | --- |
| Direct referral to external support services during a personal conversation with the patient |  |  |
| Own consultation in the practice during a personal conversation with the patient |  |  |
| Referral to institutions to which the patient initiates contact |  |  |
| Providing flyers/addresses to patients |  |  |
| Referral to an external link worker |  |  |
| An institutionalized contact person already exists in the practice |  |  |
| Other, namely: |  |  |

| Meaningfulness of SP | Yes | No |
| --- | --- | --- |
| Do you think social prescribing is meaningful? |  |  |

| Please evaluate the following statements regarding social prescribing outcomes | Does not apply | Rather does not apply | Rather applies | Applies |
| --- | --- | --- | --- | --- |
| Social prescribing leads to an improvement in… |  |  |  |  |
| Patient care |  |  |  |  |
| Patient satisfaction |  |  |  |  |
| Feelings of loneliness in patients |  |  |  |  |
| Mental health of patients |  |  |  |  |
| Social prescribing leads to a reduction in… |  |  |  |  |
| Number of consultation reasons in your own practice |  |  |  |  |
| Your own workload |  |  |  |  |
| Number of consultation reasons in other healthcare facilities |  |  |  |  |
| Number of medication prescriptions |  |  |  |  |


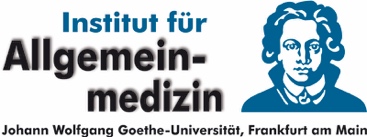


|  | Less than once a day | Once a day | 2–3 times a day | More than 3 times a day |
| --- | --- | --- | --- | --- |
| How often are you confronted with the social problems of your patients in a week? |  |  |  |  |

|  | Less than 10% | 10–20% | 20–30% | More than 30% |
| --- | --- | --- | --- | --- |
| How much of your working time as a general practitioner is spent on the social concerns of your patients? |  |  |  |  |

| Which social issues occur most frequently in your practice? | Never | Rarely | Often | Very often |
| --- | --- | --- | --- | --- |
| Loneliness |  |  |  |  |
| Workplace burdens/unemployment |  |  |  |  |
| Financial problems/poverty |  |  |  |  |
| Discrimination/exclusion |  |  |  |  |
| Issues in education/training |  |  |  |  |
| Dispute with a close person |  |  |  |  |
| Caregiving for relatives |  |  |  |  |
| Illness or death of relatives or friends |  |  |  |  |
| Mental strain/overwhelm |  |  |  |  |
| Problems with housing/homelessness |  |  |  |  |
| Social legal issues |  |  |  |  |
| Abuse/domestic violence |  |  |  |  |

| In which social issues do you think social prescribing can achieve the greatest benefit? | None | Low | Moderate | High |
| --- | --- | --- | --- | --- |
| Loneliness |  |  |  |  |
| Workplace burdens/unemployment |  |  |  |  |
| Financial problems/poverty |  |  |  |  |
| Discrimination/exclusion |  |  |  |  |
| Issues in education/training |  |  |  |  |
| Dispute with a close person |  |  |  |  |
| Caregiving for relatives |  |  |  |  |
| Illness or death of relatives or friends |  |  |  |  |
| Mental strain/overwhelm |  |  |  |  |
| Problems with housing/homelessness |  |  |  |  |
| Social legal issues |  |  |  |  |
| Abuse/domestic violence |  |  |  |  |

| Which type of referral to social care structures would you, as a general practitioner, like to use? | Does not apply | Rather does not apply | Rather applies | Applies |
| --- | --- | --- | --- | --- |
| Referral of patients to an external link worker |  |  |  |  |
| Institutionalized contact person/link worker (e.g., social worker) |  |  |  |  |
| MFA or nursing staff with additional link worker function |  |  |  |  |
| Online platform with listed social prescribing activities/cooperation partners from the third sector |  |  |  |  |
| Hotline through which programs for patients are referred – you, as the physician, establish contact |  |  |  |  |
| Providing information/addresses to various institutions – the patient establishes contact themselves |  |  |  |  |
| Referral to telephone counseling services – the patient establishes contact themselves |  |  |  |  |
| Direct contact by the general practitioner with institutions |  |  |  |  |


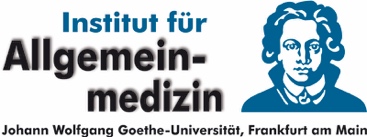


| Satisfaction of supporting patients | Yes | No |
| --- | --- | --- |
| Are you satisfied with your ability to support your patients in social matters? |  |  |

| Implementation wish of SP | Yes | No |
| --- | --- | --- |
| Would you like to implement social prescribing in your daily practice? |  |  |

| What do you currently see as the biggest barriers to implementing social prescribing? | Does not apply | Rather does not apply | Rather applies | Applies |
| --- | --- | --- | --- | --- |
| Lack of time during consultations |  |  |  |  |
| Funding for additional staff |  |  |  |  |
| Additional workload for already employed staff |  |  |  |  |
| Lack of knowledge about available regional services |  |  |  |  |
| Lack of structures for referrals |  |  |  |  |
| Lack of reimbursement for extended physician consultations |  |  |  |  |
| Lack of consensus in the practice team (multiprofessionality) |  |  |  |  |
| Limited availability/lack of space for link workers |  |  |  |  |
| Lack of understanding of social prescribing/link working |  |  |  |  |

**Supplementary Material 2. Tables research question 5**

Table 2. PERMANOVA result of testing differences between facilitators and barriers regarding self-reported SP use (yes vs. no)

|  | Df | Sum of Squares | Mean of Squares | Pseudo F-value | p-value |
| --- | --- | --- | --- | --- | --- |
| Self-reported SP usage | 1 | 40.24 | 40.241 | 3.068 | .037 |
| Residuals | 99 | 1298.63 | 13.117 | - | - |
| Total | 100 | 1338.87 | - | - | - |

Table 3. Binary logistic regression analysis of factors associated with self-reported adoption of SP

|  | Regression coefficient (β) | Standard error | p-value | Odds ratio (95% CI) |
| --- | --- | --- | --- | --- |
| Male (vs Female) | 0.749 | 0.446 | .093 | 2.11 (0.88-5.06) |
| Practice location < 5000 inhabitants * | 1.106 | 0.505 | .028 | 3.02 (1.13-8.13) |

*vs >5000 – 20,000 inhabitants **Supplementary Figures**

**Supplementary Figure 1. Frequency of Social Issues in Practice**

**Supplementary Figure 2. Perceived Benefit of SP for Social Issues in Practice**

**Supplementary Figure 3. Perceived Benefits of SP**

**Supplementary Figure 4. Perceived Barriers of SP**
